# Protein-bound polysaccharide from *Phellinus linteus *inhibits tumor growth, invasion, and angiogenesis and alters Wnt/β-catenin in SW480 human colon cancer cells

**DOI:** 10.1186/1471-2407-11-307

**Published:** 2011-07-22

**Authors:** Kyoung-Sub Song, Ge Li, Jong-Seok Kim, Kaipeng Jing, Tae-Dong Kim, Jin-Pyo Kim, Seung-Bo Seo, Jae-Kuk Yoo, Hae-Duck Park, Byung-Doo Hwang, Kyu Lim, Wan-Hee Yoon

**Affiliations:** 1 Department of Biochemistry, College of Medicine, Chungnam National University, Joong-gu, Daejeon 301-747, Korea; 2 Cancer Research Institute, Chungnam National University, Joong-Ku, Daejeon 301-747, Korea; 3 Infection Signaling Network Research Center, Chungnam National University, Joong-Ku, Daejeon 301-747, Korea; 4 Department of General Surgery, Yanbian University Hospital, Jilin 133000, People's Republic of China; 5 College of Pharmacy, Chungnam National University, Daejeon 301-747, Korea; 6 Ja Kwang Research Institute, Hankook Sin Yak Pharmaceutical Company, Nonsan 320-854, Korea; 7 Dr. Park's Breast Clinic, Daejeon, Korea

## Abstract

**Background:**

Polysaccharides extracted from the *Phellinus linteus *(PL) mushroom are known to possess anti-tumor effects. However, the molecular mechanisms responsible for the anti-tumor properties of PL remain to be explored. Experiments were carried out to unravel the anticancer effects of PL.

**Methods:**

The anti-cancer effects of PL were examined in SW480 colon cancer cells by evaluating cell proliferation, invasion and matrix metallo-proteinase (MMP) activity. The anti-angiogenic effects of PL were examined by assessing human umbilical vein endothelial cell (HUVEC) proliferation and capillary tube formation. The *in vivo *effect of PL was evaluated in an athymic nude mouse SW480 tumor engraft model.

**Results:**

PL (125-1000 μg/mL) significantly inhibited cell proliferation and decreased β-catenin expression in SW480 cells. Expression of *cyclin D1*, one of the downstream-regulated genes of β-catenin, and T-cell factor/lymphocyte enhancer binding factor (TCF/LEF) transcription activity were also significantly reduced by PL treatment. PL inhibited *in vitro *invasion and motility as well as the activity of MMP-9. In addition, PL treatment inhibited HUVEC proliferation and capillary tube formation. Tumor growth of SW480 cells implanted into nude mice was significantly decreased as a consequence of PL treatment, and tumor tissues from treated animals showed an increase in the apoptotic index and a decrease in β-catenin expression. Moreover, the proliferation index and microvessel density were significantly decreased.

**Conclusions:**

These data suggest that PL suppresses tumor growth, invasion, and angiogenesis through the inhibition of Wnt/β-catenin signaling in certain colon cancer cells.

## Background

Colorectal cancer (CRC) is the third most prevalent form of cancer in men and women, with a 5-year survival rate of 63%, decreasing to 10% in patients with metastatic disease [1]. Thus, the formation of distant metastasis is the decisive and most lethal event during the course of the disease. Although recent advances in chemotherapeutic agents in CRC have been achieved, treatment options are still limited and are associated with significant morbidity and mortality.

Mushroom polysaccharides are widely being used as nonspecific immunostimulants for cancer patients in Asian countries. The Polysaccharide isolated from *Phellinus linteus *(PL) is an immunomudulatory agent with a molecular weight of 153 kDa [2]. PL stimulates the proliferation of T lymphocytes and activates B cells [3], induces secretory and celluar macrophage response [4], and inhibits tumor growth and metastasis through the immunopotentiation [5]. It had been suggested that antitumor effect are not only immunomodulatory, but may result from a direct action on tumor cells.

We previously demonstrated that PL has an antiproliferative effect for SW480 colon cancer cells and the growth inhibition is mediated by induction of apoptosis and G_2_/M cell cycle arrest which are associated with decrease in Bcl-2, increase of the release of cytochrome *c*, and reduced expression of cyclin B1 [6]. Since our report, the direct antitumor effect of PL has been demonstrated by others: the inhibition of pulmonary metastasis of melanoma cells through the downregulation of mRNA level of urokinase plasminogen activator (uPA) [7], suppressed proliferation of lung cancer cells by the inhibition of cyclin-dependent kinases cdk2, 4, and 6, and induced apoptosis through the activation of caspase 3 [8], apoptosis of prostate cancer cells [9,10], and inhibition of tumor growth and invasive behavior of breast cancer cells mediated by cell cycle arrest at S phase and inhibition of serine-threonine kinase AKT signallig [11].

One important signaling pathway involved in the etiology of colon cancer is Wnt/β-catenin, and more than 90% of colon cancers bear mutations that result in the activation of this pathway [12]. Activating mutations in genes of the Wnt/β-catenin signaling pathway are observed early in the development of colon cancers. Mutations that activate the Wnt/β-catenin pathway generally affect β-catenin phosphorylation and stability [13]. Phosphorylated β-catenin is degraded via the ubiquitin pathway. In the absence of efficient degradation such as genetic mutations of adenomatous polyposis coli (APC) or β-catenin, β-catenin accumulates and is transported to the nucleus, where it acts as a transcription factor in concert with T-cell factor/lymphocyte enhancer binding factor (TCF/LEF) [14,15]. The resulting β-catein-TCF/LEF complex activates TCF target genes which affect cell proliferation, survival, angiogenesis, invasion and metastasis [16]. Recent evidences suggested that although mutations of components of the Wnt/β-catenin pathway generally occur early in colon cancer progression, accumulation of β-catenin in the nucleus has been associated with late stages of tumor progression and the development of metastasis [17-19].

In the present study, we have investigated the effects of a PL treatment on multiple steps involved in colon cancer growth, invasion and neoangiogenesis. Herein, we show that PL inhibits proliferation, motility and invasion as well as matrix metalloproteinases (MMPs) and tumor neoangiogenesis of SW480 colon cancer cells *in vitro *and *in vivo*. Furthermore, we demonstrated that PL significantly inhibited Wnt/β-catenin signaling pathway.

Our data suggest that the PL-induced down-regulation of Wnt/β-catenin signaling may contribute to the inhibition of tumor growth, invasion and angiogenesis of SW480 colon cancer cells.

## Methods

### Cell lines and culture conditions

SW480 human colon cancer cells and HT1080 fibro-sarcoma cells were obtained from American Type Culture Collection (ATCC, Rockville, MD). The cells were grown in Dulbecco's Modified Eagle's Medium (DMEM) containing 10% fetal bovine serum (FBS), penicillin (100 U/mL), and streptomycin (100 μg/mL). Cultures were maintained at 37°C in a humidified 5% CO_2 _atmosphere. PL (Hankook Sin Yak Pharm., Nonsan, Korea) was dissolved in DMEM and adjusted to the indicated final concentrations with culture medium before use. Human umbilical vein endothelial cells (HUVECs) were isolated from fresh umbilical cords obtained by caesarean section by a modification of the technique previously described [20]. HUVECs were cultured in gelatin-coated plates with Endothelial Basal Medium-2 (EBM-2) supplemented with 10 mL FBS, 0.2 mL hydrocortisone, 2 mL human fibroblast growth factor-basic (hFGF-B), 0.5 mL vascular endothelial growth factor (VEGF), 0.5 mL R^3^-IGF-1, 0.5 mL ascorbic acid, 0.5 mL human epidermal growth factor (hEGF), 0.5 mL GA-1000, 0.5 mL heparin (EBM-2 Bullet kit, Clonetics) and incubated at 37°C in a humidified incubator containing 5% CO_2_. HUVECs were used at passages 2-5.

### Cell proliferation assay

The effect of PL on the growth of colon cancer cells was evaluated using 5 × 10^3 ^cells seeded onto 96-well plates (Corning, NY), which were treated with PL simultaneously at the time of cell plating. To evaluate the effect of PL at concentrations 125, 250, 500, and 1,000 μg/mL, cells were maintained in media with various concentrations of PL for up to 96 h and cell numbers were determined by a tetrazolium-based colorimetric assay (MTT, Sigma, St. Louis, MO) [21], and absorbance was read at 570 nm.

### Preparation of cell lysates and western blot analysis

Proteins were extracted with RIPA buffer (50 mM Tris-HCl, pH7.5, 150 mM NaCl, 5 mM ehylenediaminetetraacetic acid [EDTA], 1% Nonidet P-40, 0.1% sodium dodecyl sulphate [SDS], and 1% sodium deoxycholate) containing protease inhibitor cocktail tablets (Roche Diagnostics Indianapolis, IN). Samples were resolved through a 10% SDS-polyacrylamide gel and transferred to Hybond ECL membranes (Amersham Pharmacia Biotech, Buckinghamshire, UK). Membranes were blocked in 1× TBS (1 L of 10× TBS was prepared by mixing 24.2 g of Trizma base and 80 g NaCl, and the pH was adjusted to 7.6), containing 0.1% Tween 20 with 5% non-fat skim milk, for 1 h at room temperature and incubated with a primary antibody for 1 h at room temperature. After 3 washes (5 min each) in TBST (TBS containing 0.1% Tween 20), the membranes were incubated with horseradish peroxidase-conjugated secondary antibody for 1 h at room temperature. After 3 washes (5 min each) in TBST, proteins were visualized using the enhanced chemiluminescence method (Amersham Pharmacia Biotech, Buckinghamshire, UK).

### Immuno-cytochemical analysis

Cells were grown to 60% confluence on 12-well chamber slides. The cells were treated with PL for 24 h before simultaneous paraformaldehyde fixation and permeabilisation with Triton X-100. After blocking with 1% bovine serum albumin (BSA), the cells were incubated with anti-β-catenin antibody (Santa Cruz Biotechnolgy, CA) overnight at 4°C. The cells were labeled with Alexa Fluor 488-conjugated anti-mouse secondary antibody (Molecular Probes, Eugene, Oregon, USA). The slides were covered with a mounting solution (Dako, Carpinteria, CA) and photos were obtained using an LSM5 confocal microscope (Carl Zeiss, Inc., Jena, Germany).

### Luciferase reporter activity

Cells were seeded and allowed to achieve 80% confluence in 6-well plates. The cells were transiently transfected with 1 μg per well of TCF/LEF-Luc by using Lipofectamine Plus transfection reagents (Invitrogen, Carlsbad, CA), according to the manufacturer's instructions. After transfection, the cells were treated with various concentrations of PL for 24 h. Cell lysates were prepared using 1× reporter lyses buffer (Promega, Madison, WI). Luciferase activity was measured as previously described, by using an AutoLumat LB953 Luminometer (Berthold, Stevenage, UK) and using the luciferase assay system from Promega [22,23]. The relative luciferase activity was calculated after normalization of cellular proteins. All values are expressed as the percentage of activity relative to basal activity.

### *In vitro *invasion and motility assay

Transwell culture chambers containing polycarbonate filters of diameter 6.5 mm and pore size 8 μm (Costar, Cambridge, MA) were used for the assay according to a previously described method [24]. For the invasion assay, filters coated with Matrigel (160 μg per filter) were used. To investigate the effect of PL on invasion, we added various concentrations of PL to the media. After 72 h of incubation, cells on the top of the filter, which was generated by non-invasive cells, were removed using cotton swabs. The filters were removed, and the invasive cells beneath the filters were stained with Gill's hematoxylin and counted under a microscope. For the motility assay, the same system was used, but the Matrigel coating was omitted.

### Protease analysis by substrate-embedded gel

For zymography analyses [25], cells grown to 80% confluence were washed 3 times with calcium-magnesium-free phosphate-buffered saline (PBS) and cultured in DMEM without FBS. Conditioned media (CM) were obtained after 24 h of culture and centrifuged at 3,000 × *g *for 10 min to remove cells and debris. Cell-free CM was concentrated approximately 10-fold by using a Centricon-10 device (Amicon, Beverly, MA), and aliquots of the concentrated CM were normalized for cell number. Proteins in the normalized CM were then separated by electrophoresis on 10% polyacrylamide gels impregnated with 1 mg/mL gelatin (Fisher Chemical Co., Fair Lawn, NJ) or 1 mg/mL casein (Sigma Chemical Co.) containing 13 μg/mL plasminogen (Sigma Chemical Co.) under non-reducing conditions. After electrophoresis, the gels were washed twice in 2.5% Triton X-100 for 30 min, proteolysed with a reaction buffer (50 mM Tris-HCl, 5 mM CaCl_2_, and 0.02% NaN_3 _[pH 8.0]) for 72 h at 37°C, and stained with Coomassie Brilliant Blue G-250. To investigate the effect of PL on proteases, we added various concentrations of PL to the incubation buffer.

### HUVEC proliferation and capillary tube formation on matrigel

Growth assays of HUVECs were carried out according to the procedure described by Bae et al. [24], with some modifications. Briefly, HUVECs were seeded in 0.2% gelatin-coated wells in a 96-well culture plate (Corning, NY) at an initial density of 5 × 10^3 ^cells/well in 200 μL of EBM-2 and then grown under standard conditions at 37°C in 5% CO_2_. On the next day, 125, 250, 500, and 1,000 μg/mL of PL were added to each well for 3 d, and the number of viable cells was measured using the MTT assay. To further assess the anti-angiogenic effect of PL, we performed vascular tube formation experiments. HUVECs were seeded at a density of 10^4 ^cells/well in Matrigel-coated 24-well plates and incubated at final PL concentrations of 250, 500, and 1,000 μg/mL. During these incubations, HUVEC morphological changes were monitored using an inverted phase-contrast microscope (Model IX 70; Olympus, Tokyo, Japan) and photographs were obtained.

### Nude mice tumourigenicity

All animal-related procedures were reviewed and approved by the Institutional Animal Care and Use Committee of Chungnam National University. Confluent colon cancer cell cultures were harvested by brief trypsinization, washed 3 times with calcium and magnesium-free PBS, and re-suspended at a final concentration of 5 × 10^7 ^cells/mL in serum-free DMEM. Single-cell suspensions were confirmed by phase-contrast microscopy, and cell viability was determined using trypan blue exclusion; only single-cell suspensions with a viability > 90% were used. Pathogen-free female BALB/cAnNCrj-nu athymic nude mice (4 weeks old; Charles River Laboratories, Kanazawa, Japan) were anesthetized with diethyl ether by inhalation, and 5 × 10^6 ^SW480 cells in serum-free DMEM were inoculated subcutaneously (s.c.) into the right flank. From the day of tumor cell inoculation, the mice received a daily intratumoral injection (i.t.) of PL (100 μg/100 μL of saline) or intravenous injection (i.v.) of PL (25, 50, 100 μg/100 μL of saline) as well as the same amount of physiologic saline as a control for 14 d. The mice were regularly examined, tumor sizes were measured with a caliper, and tumor volumes were determined using the following formula: volume = 0.5 × (width)^2 ^× length [26]. Each experimental group consisted of 8 animals.

### Immuno-histochemical analysis

The mice were euthanized and tumors were removed and bisected. One part of the tumor was placed in neutral buffered formalin for paraffin block preparation, and the other part was frozen for preparation of cryo-cut sections. The degree of apoptosis was evaluated using an ApopTag apoptosis detection kit (S7101; Intergen, Norcross, GA), according to the manufacturer's recommendations. The apoptotic index was calculated as the percentage of nuclei stained by peroxidase, showing nuclear halo or apoptotic bodies. Positive cells were quantified by counting the number of brown-stained nuclei/total number of cells in 5 randomly selected 400× magnified fields.

To evaluate the proliferation index, the paraffin sections were incubated with a monoclonal mouse Ki-67 antibody (MIB-1; Dako, Carpinteria, CA) at a dilution of 1:100. Staining was carried out with a universal labeled streptavidin-biotin kit (Dako, Carpinteria, CA), according to the standard protocol. The proliferation index was determined by counting stained cells at 400×. To immuno-localize tumor blood vessels, the cryo-sections were stained with a monoclonal rat anti-mouse CD31 antibody (PECAM-1; BD PharMingen, San Diego, CA) at a dilution of 1:50. The antigen-antibody reaction was visualized using an anti-mouse immunoglobulin horseradish peroxidase detection kit (BD PharMingen, San Diego, CA), according to the manufacturer's recommendations. Vessel density was determined by counting the Positive cells were quantified by counting the standard vessels in 5 randomly selected 200× magnified fields.

To confirm tissue β-catenin levels, paraffin sections were de-parafinised in xylene and dehydrated in serially diluted ethanol. Antigen retrieval was performed using citrate buffer (pH 6.0). The sections were blocked with a protein blocker (Dako, Carpinteria, CA) and stained with an anti-β-catenin antibody (Santa Cruz Biotechnology, CA). The sections were then stained with hematoxylin and photographed under a light microscope. Positive cells were quantified by counting the number of brown-stained cells/total number of cells in 5 randomly selected 400× magnified fields

### Statistical analysis

Data are expressed as mean and standard deviation (SD), and significance was established by unpaired Student's *t *test. For all analyses, the level of statistical significance was more than the 95% confidence level (P < 0.05). *, **, or *** indicates *P *< 0.05, *P *< 0.01, or *P *< 0.001, respectively.

## Results and Discussion

### PL inhibited cell proliferation and Wnt/β-catenin signaling pathway activity in SW480 colon cancer cells *in vitro*

Although the growth inhibitory effect of PL has been reported in different types of cancers, including colorectal cancer [4-11], the molecular mechanisms responsible for the anti-tumor and anti-invasive behavior of PL remain elusive.

**Figure 1:**
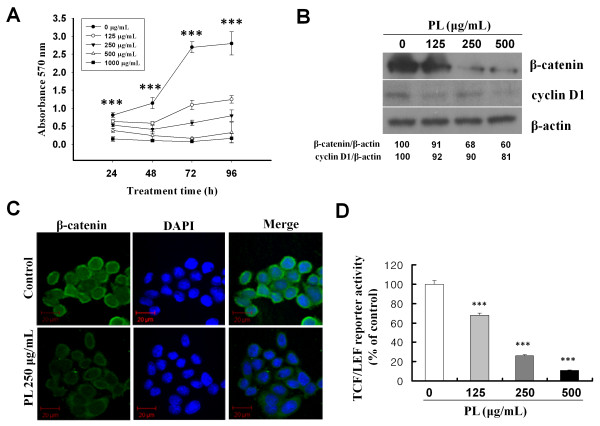
**PL inhibited cell growth and Wnt/β-catenin signaling in SW480 human colon cancer cells**. A, SW480 cells were treated with various concentrations of PL for the indicated times. At the indicated time points, cell growth was measured by the MTT assay as described in Methods. The data represent the mean of three independent experiments ± SD. ***, P < 0.001 versus control. B, Cells were treated with various concentrations of PL (125-500 μg/mL). After 24 h, protein samples were prepared and each sample was electrophoresed in an SDS-PAGE gel and immuno-blotted to detect β-catenin and cyclin D1. The blots shown are representative of three independent experiments with similar results. C, SW480 cells were treated with PL (250 μg/mL) and immuno-stained for β-catenin. Representative images were obtained by confocal microscopy. D, SW480 cells transiently transfected with p-luc TCF/LEF and treated with the indicated concentrations of PL. Luciferase reporter activity was measured in cell extracts after 16 h of PL treatment. Error bars, SD (n = 3). ***, P < 0.001 versus control.

SW480 colon cancer cells, which are characterized by β-catenin over-expression and a mutant APC (25), were used to evaluate the effect of PL on cell proliferation by the MTT assay. PL was added to the SW480 cell culture medium at the time of plating and maintained for 24, 48, 72, and 96 h. Treatment with PL (125-1,000 μg/mL) resulted in a significant dose-dependent inhibition of cell proliferation (Figure 1A, P < 0.001). Furthermore, cell proliferation was almost ceased when 250-1,000 μg/mL of PL was added. These results indicate that PL inhibits the proliferation of SW480 cells, as previously reported [6].

The intracellular levels of β-catenin are tightly regulated by its degradation complex composed of APC, glycogen synthase kinase-3β (GSK-3β), and Axin [27]. Moreover, APC controls the sub-cellular localization of β-catenin by nuclear-cytoplasmic shuttling [28]. Loss of functional APC protein leads to the inappropriate stabilization of β-catenin [29]. In virtually all cases of colon cancer, mutations target components of the Wnt/β-catenin signaling pathway [30]. We examined the potential effect of PL on β-catenin protein level and activity in SW480 cells, since these cells are reported to carry mutations of APC and over-express β-catenin [31]. As shown in Figure 1B, treatment with PL reduced the levels of β-catenin protein as well as cyclin D1, a downstream-regulated gene of β-catenin, in a dose-dependent fashion. This result indicates that PL effectively inhibits the accumulation of β-catenin and the expression of its downstream genes.

The functions of β-catenin are determined by its sub-cellular distribution. In general, β-catenin localizes to the cytoplasm or nucleus with a preference for the peri-plasma membrane, where it binds to E-cadherin. In cells over-expressing β-catenin, as in the majority of colon cancer cells, the protein is predominantly present in a de-phosphorylated form and translocates into the nucleus, activating the transcription of target genes [30]. In the present study, the effect of PL on the intracellular localization of β-catenin was evaluated using indirect immuno-fluorescence analysis after PL treatment. Under control conditions, β-catenin was detected in the cytoplasm and nucleus, but preferentially accumulated in the nucleus. Cells treated with PL showed a remarkable decrease of β-catenin staining in both cytoplasm and nucleus (Figure 1C).

Because β-catenin regulates gene expression by forming a complex with TCF/LEF transcription factor family proteins and binding to the promoter region of target genes, we further examined the effect of PL on TCF/LEF reporter activity. TCF/LEF transcriptional activity was assayed after transient transfection of a luciferase reporter construct under the control of a TCF/LEF response element. As shown in Figure 1D, PL treatment significantly inhibited TCF/LEF reporter activity in a dose-dependent fashion (P < 0.001). This result strongly suggests that PL modulates the Wnt/β-catenin signaling pathway.

### PL inhibited the invasion and motility of SW480 cells and the secretion of MMP-2 and MMP-9 from SW480 cells

**Figure 2:**
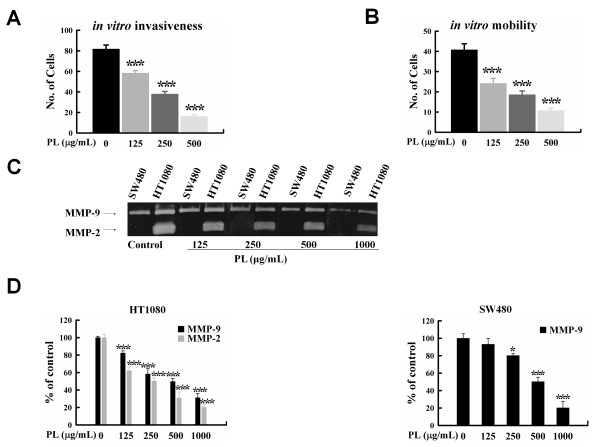
**PL inhibited *in vitro *invasion and motility of SW480 cells and the activity of MMP-9**. A, The histogram shows the mean number of invasive cells for a 20× field of view. Counts were performed on three inserts and five fields of view per insert. Cells were loaded onto Matrigel-coated upper chambers of Transwell plates and treated with the indicated concentrations of PL; filtrated cells were stained. The error bars represent the SD of the mean number of invasive cells per field of view for all fields. ***, P < 0.001 versus control. B, Cells were loaded onto gelatin-coated upper chambers, and filtrated cells were stained. The histogram shows the mean number of invasive cells for a 20× field of view. Counts were performed on three inserts and five fields of view per insert. The error bars represent the SD of the mean number of invasive cells per field of view for all fields. ***, P < 0.001 versus control. C, Conditioned media was prepared from SW480 and HT1080 cells and electrophoresed in a gel containing gelatin. The gels were incubated for 24 h with the indicated concentrations of PL. The gel shown is representative of three independent experiments with similar results. D, The densities of bands of MMP-2 and MMP-9 were quantified by imaging densitometry. MMP-2 and MMP-9 activity of the control was set at 100%; the activities of PL-treated samples are represented as percentages of the control. *, P < 0.05 versus control; ***, P < 0.001 versus control.

Tumor cell invasion and metastasis are multistep phenomena involving the proteolytic degradation of the basement membrane and extracellular matrix (ECM), altered cell adhesion, and physical movement of tumor cells [20]. Although the correlation between β-catenin expression patterns and clinical outcome is somewhat a controversial subject, there are studies reporting that positive nuclear expression of β-catenin at the invasive front of colorectal carcinomas predicts shorter survival [32,33]. Current studies provide evidence that activation of Wnt/β-catenin signaling is associated with increased expression of MMPs, which are key enzymes involved in invasion and metastasis, in endothelial cells [34], chondrocytes [35], and cancer cells [36,37]. To determine whether PL affects the invasive ability of human colon cancer cells *in vitro*, we performed invasion and motility assays by using various concentrations of PL. Treatment with 125, 250, and 500 μg/mL of PL significantly inhibited invasion of SW480 cells in a dose-dependent manner (28.6%, 53.5%, and 80.2%, respectively; P < 0.001; Figure 2A). In addition, cell motility was significantly inhibited by increasing PL concentrations (40.7%, 54.4%, and 73.5% inhibition with 125, 250, and 500 μg/mL of PL, respectively; P < 0.001; Figure 2B).

The key enzymes that have been shown to be closely associated with invasive and metastatic potential are MMPs and uPA [38,39]. Several studies have shown that PL inhibits cancer cell invasion and metastasis by activating host immunity [40-42]. Recently, PL was shown to suppress invasiveness through the inhibition of uPA secretion in mouse melanoma cells and breast cancer cells [7,11]. To evaluate the inhibitory effect of PL on MMPs and uPA, the CM of SW480 colon cancer cells and HT1080 fibro-sarcoma cells (as a control for MMP-9 and MMP-2) were evaluated zymographically. However, we did not observe any effect of PL on uPA activity in a casein and plasminogen-impregnated gel system (data not shown). Moreover, PL treatment did not affect MMP gene expression or secretion as indicated by reverse transcriptase-polymerase chain reaction and gelatin zymography (data not shown). Interestingly, PL inhibited the activity of MMPs without affecting their expression or secretion. Treatment with 500 and 1,000 μg/mL of PL significantly reduced MMP-9 and MMP-2 activities in SW480 and HT1080 cells (Figure 2C and 2D). Although the exact mechanism of PL inhibition of MMP-9 and MMP-2 activities is not clear, this finding provides the first evidence of the potential effect of PL on the direct inhibition of these gelatinolytic activities. Taken together, the inhibitory effects of PL on cellular invasion, *in vitro *motility, and the activity of MMPs suggest that PL has a direct anti-invasive effect on certain colon cancer cells.

### Effect of PL on HUVEC growth and capillary tube formation

**Figure 3:**
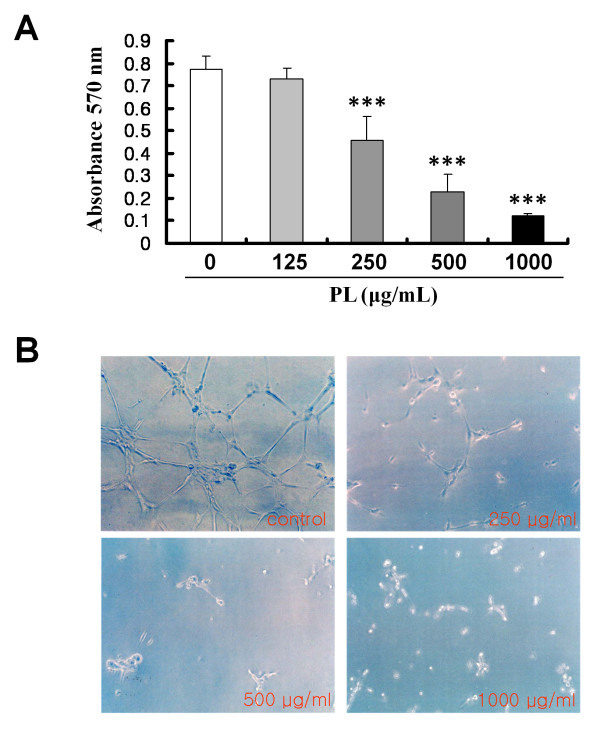
**Effect of PL on proliferation of HUVECs and capillary tube formation**. A, HUVECs were seeded in 0.2% gelatin-coated wells of 96-well plates at an initial density of 5 × 10^3 ^cells/well. On the next day, the indicated concentrations of PL were added to each well for 24 h, and cell numbers were determined using the MTT assay as described in Methods. The data shown represent the mean ± SD of 3 independent experiments (***, *P *< 0.001). B, PL inhibited capillary tube formation by HUVECs on Matrigel. HUVECs were seeded at a density of 10^4 ^cells/well in 1:2-diluted Matrigel-coated 24-well plates and incubated with the indicated concentrations of PL. At 18 h, the control HUVECs had formed an interconnected network of anastomosing cells (which had a honeycomb appearance), whereas HUVECs treated with PL showed a significant reduction in tube formation in a dose-dependent manner. Photomicrographs were taken using an inverted phase-contrast microscope.

Tumor angiogenesis is critical for the growth and metastasis of solid tumors [24]. Angiogenesis is both complex and dynamic and requires the proliferation of endothelial cells from pre-existing blood vessels, breakdown of the ECM, and migration of endothelial cells [20,43]. To elucidate the anti-angiogenic effect of PL, HUVEC proliferation and tube formation were investigated. The number of HUVECs cultured on gelatin was examined by MTT colorimetric assay to determine the cytotoxic effect of PL. In this experiment, PL was found to have a significant dose-dependent cytotoxic effect on the proliferation of HUVECs. The rate of growth inhibition was 5.2, 40.9, 70.4, and 84.5% at 125, 250, 500, and 1,000 μg/mL of PL, respectively (250, 500, 1000 μg/mL, P < 0.001; Figure 3A).

The morphological features of HUVECs were also evaluated on Matrigel-coated plates after treatment with various concentrations of PL. Eighteen hours post-treatment, control HUVECs had formed an interconnected network of anastomosing cells, which under a low-power light microscope had a 'honeycomb' appearance (Figure 3B; control). However, this interconnected network, which resembled a vessel-like structure, progressively disappeared with increasing PL concentrations (Figure 3B; 250-1,000 μg/mL of PL). These results are consistent with a recent observation that PL inhibited capillary morphogenesis of human aortic endothelial cells [11] and angiogenesis in a CAM chick embryo assay [44].

### Effect of PL on SW480 human colon cancer cell growth *in vivo*

**Figure 4:**
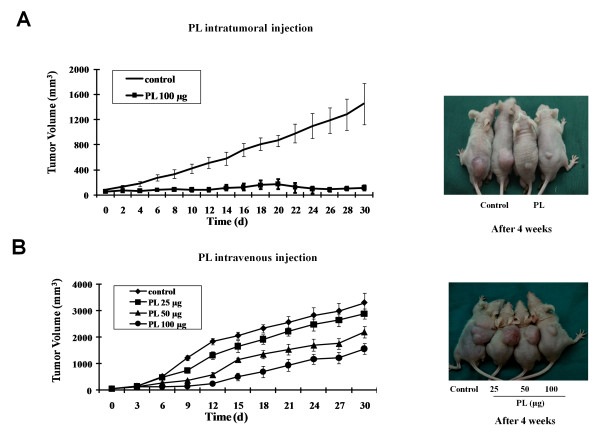
**Effect of PL on the growth of human colon cancer cells in nude mice**. SW480 colon cancer cells (5 × 10^6 ^cells/100 μL) were injected s.c. into the right flank of the nude mice. From the day of inoculation, the mice were administered a daily i.t. injection of (A) PL (100 μg/100 μL of saline) or i.v. injection of  (B) PL (25-100 μg/100 μL of saline). Control mice were injected with the same amount of saline until 1 month after tumor cell implantation. Tumor size was measured with a caliper at the indicated times. The greatest dimension of the tumor and the 1 perpendicular to it were measured using the caliper and tumor size was calculated as 0.5 × (width)^2 ^× length = tumor volume.

We showed that PL significantly inhibits SW480 cell proliferation *in vitro *in a dose-dependent manner. Furthermore, treatment with PL down-regulated β-catenin and cyclin D1 expressions in SW480 cells *in vitro*. To evaluate whether PL affects the proliferation of SW480 cells *in vivo*, we performed a tumourigenicity assay in nude mice. Daily i.t. treatment with PL (100 μg/100 μL of saline) for 2 weeks resulted in a significant reduction in SW480 tumor volume compared with saline-treated controls (74.0%, 80.9%, 86.9%, and 92.1% reduction at 1-, 2-, 3-, and 4-week post-inoculation, respectively; P < 0.001, Figure 4A). Interestingly, PL-treated tumors appeared to be in a dormant state during the entire experimental period of 30 d, even 16 d after PL treatment was discontinued (days 16-30). The effect of PL on SW480 tumor growth in nude mice was also evaluated using different routes of administration and various doses (25-100 μg per mouse) for 14 d from the day of tumor inoculation. Daily i.v. PL treatment at 50 and 100 μg for 14 d significantly reduced SW480 tumor volume. Tumor volume was reduced by 47.5% and 73.4% at 1 week, 44.0% and 75.5% at 2 weeks, 39.9% and 58.4% at 3 weeks, and 33.2% and 52.5% at 4 weeks with PL concentrations of 50 and 100 μg per mouse, respectively (P < 0.001, Figure 4B). However, treatment with a low dose of PL (25 μg/mice) significantly reduced tumor volume only after a 2-week treatment period (P < 0.05). It is worth noting that the tumor volume in mice received i.t. treatment of PL (100 μg/d) appears to be smaller than that in mice received i.v. injections (100 μg/d), indicating that direct i.t. administration of PL is more effective in inhibiting tumor growth, possibly due to a relatively high concentration or prolonged release of PL in tumor tissues resulted from i.t. administration. Together, these results strongly indicate that PL inhibits tumourigenicity of colon cancer cells.

### Immuno-histochemical analysis of proliferation, apoptosis, angiogenesis, and β-catenin expression in SW480 tumor tissues

**Figure 5:**
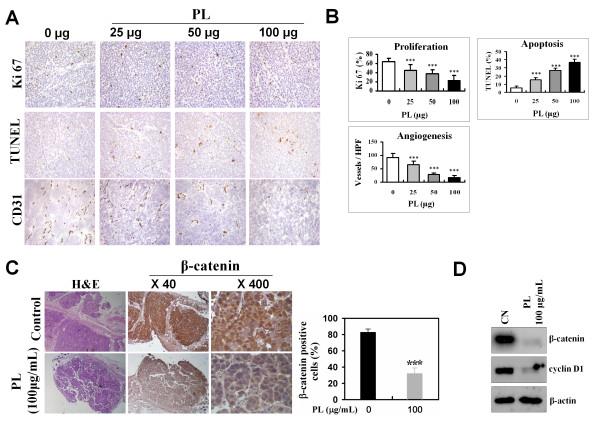
**Immuno-histochemical analysis of SW480 tumors in the nude mice treated with PL**. The nude mice were inoculated with SW480 cells (5 × 10^6 ^cells/100 μL) and administered daily i.v. injections of PL (100 μg/100 μL of saline) or normal saline for 14 d after tumor cell implantation. Two weeks after the implantation, tumors were removed, and histological sections of the tumors from saline- or PL-treated mice were processed for immuno-histochemical staining and quantified for proliferation (Ki-67), apoptosis (TUNEL), and angiogenesis (CD31). A, Immuno-histochemical staining for proliferation, apoptosis, and angiogenesis. B, Quantification of proliferation, apoptosis, and angiogenesis index. The quantitative data in each case represent the mean ± SD of 8 animals in each group as detailed in section Methods. C, SW480 tumor tissue samples were immuno-histochemically stained for β-catenin immuno-reactivity (left) and quantified (right) as detailed in Methods. Quantitative data in each case represent the mean ± SD of 8 animals in each group. D, Expression of β-catenin and cyclin D1 in SW480 tumors. Total protein was prepared from saline- or PL-treated tumor tissues and electrophoresed in an SDS-PAGE gel followed by immuno-blotting for β-catenin and cyclin D1. The blots shown are representative of three independent experiments with similar results.

Histological sections of the SW480 tumor tissues grown in nude mice for 14 d were analyzed for proliferation, apoptosis, neo-vascularization, and β-catenin expression (Figure 5A and 5C). Immuno-histochemical analysis of cell proliferation (Ki-67) showed a significant dose-dependent reduction in PL-treated tumors compared with control tumors (29.3%, 40.3%, and 65.3% reduction with 25, 50, and 100 μg PL, respectively; P < 0.001, Figure 5B, Ki-67). The apoptotic index was significantly increased in PL-treated tumors (1.8-, 4.9-, and 6.7-fold higher than control tumors with 25, 50, and 100 μg of PL, respectively; P < 0.001, Figure 5B, Apoptosis). Furthermore, neo-angiogenesis in PL-treated tumors, as determined by the number of CD31-stained microvessels, was significantly lower than in control tumors (28.7%, 68.3%, and 80.5% reduction at 25, 50, and 100 μg of PL, respectively; P < 0.001, Figure 5B, Angiogenesis).

We previously showed that β-catenin is preferentially accumulated in the cytoplasm and nucleus of SW480 cells, and PL treatment significantly decreases the amount of β-catenin protein. To confirm this finding *in vivo*, we evaluated the changes in the levels of β-catenin protein in SW480 tumor tissues in response to PL treatment. As shown in Figures 5C, PL (100 μg/mL) treatment markedly decreased the level of cytoplasmic as well as nuclear β-catenin protein in SW480 tumor tissues. Moreover, the levels of β-catenin and cyclin D1 protein were also significantly reduced in the PL-treated tumors (Figure 5D). These results are consistent with the data obtained in our *in vitro *experiments.

## Conclusions

In conclusion, the present results show that PL isolated from *Phellinus linteus *causes a significant reduction in β-catenin protein levels and the down-regulation of certain downstream genes in the Wnt/β-catenin pathway in SW480 colon cancer cells *in vitro *and *in vivo*. In addition, we showed that PL significantly reduces invasiveness of SW480 cells through a direct effect on the activity of cellular MMPs, motility, and angiogenesis, which are strongly associated with Wnt/β-catenin signaling. The present data suggest that PL can be developed as an effective therapeutic agent for patients with colon cancer through its effect on the inhibition of multiple steps involved in colon cancer growth, invasion, and neo-angiogenesis by suppression of Wnt/β-catenin signaling.

## Competing interests

The authors declare that they have no competing interests.

## Authors' contributions

KSS, GL and JSK carried out the molecular studies and drafted the manuscript. KPJ helped to prepare the manuscript. TDK, JPK, SBS and JKY participated in the immunoassays and performed the statistical analysis. HDP, BDH, KL and WHY conceived of the study, and participated in its design and coordination. All authors read and approved the final manuscript.

## Pre-publication history

The pre-publication history for this paper can be accessed here:

http://www.biomedcentral.com/1471-2407/11/307/prepub

## References

[B1] Goldberg RM Advances in the treatment of metastatic colorectal cancer Oncologist 2005 10 Suppl 3 40 48 16368870 10.1634/theoncologist.10-90003-40

[B2] Song KS Cho SM Lee JH Kim HM Han SB Ko KS Yoo ID B-lymphocyte-stimulating polysaccharide from mushroom Phellinus linteus Chem Pharm Bull (Tokyo) 1995 43 12 2105 2108 10.1248/cpb.43.2105 8582012

[B3] Kim HM Han SB Oh GT Kim YH Hong DH Hong ND Yoo ID Stimulation of humoral and cell mediated immunity by polysaccharide from mushroom Phellinus linteus Int J Immunopharmacol 1996 18 5 295 303 10.1016/0192-0561(96)00028-8 8933208

[B4] Kim GY Oh YH Park YM Acidic polysaccharide isolated from Phellinus linteus induces nitric oxide-mediated tumoricidal activity of macrophages through protein tyrosine kinase and protein kinase C Biochem Biophys Res Commun 2003 309 2 399 407 10.1016/j.bbrc.2003.08.018 12951063

[B5] Han SB Lee CW Jeon YJ Hong ND Yoo ID Yang KH Kim HM The inhibitory effect of polysaccharides isolated from Phellinus linteus on tumor growth and metastasis Immunopharmacology 1999 41 2 157 164 10.1016/S0162-3109(98)00063-0 10102797

[B6] Li G Kim DH Kim TD Park BJ Park HD Park JI Na MK Kim HC Hong ND Lim K Hwang BD Yoon WH Protein-bound polysaccharide from Phellinus linteus induces G2/M phase arrest and apoptosis in SW480 human colon cancer cells Cancer Lett 2004 216 2 175 181 10.1016/j.canlet.2004.07.014 15533593

[B7] Lee HJ Lee HJ Lim ES Ahn KS Shim BS Kim HM Gong SJ Kim DK Kim SH Cambodian Phellinus linteus inhibits experimental metastasis of melanoma cells in mice via regulation of urokinase type plasminogen activator Biol Pharm Bull 2005 28 1 27 31 10.1248/bpb.28.27 15635158

[B8] Guo J Zhu T Collins L Xiao ZX Kim SH Chen CY Modulation of lung cancer growth arrest and apoptosis by Phellinus Linteus Mol Carcinog 2007 46 2 144 154 10.1002/mc.20275 17131292

[B9] Zhu T Guo J Collins L Kelly J Xiao ZJ Kim SH Chen CY Phellinus linteus activates different pathways to induce apoptosis in prostate cancer cells Br J Cancer 2007 96 4 583 590 10.1038/sj.bjc.6603595 17262078 PMC2360058

[B10] Tsuji T Du W Nishioka T Chen L Yamamoto D Chen CY Phellinus linteus extract sensitizes advanced prostate cancer cells to apoptosis in athymic nude mice PLoS One 5 3 e9885 10.1371/journal.pone.0009885 PMC2847601 20360989

[B11] Sliva D Jedinak A Kawasaki J Harvey K Slivova V Phellinus linteus suppresses growth, angiogenesis and invasive behaviour of breast cancer cells through the inhibition of AKT signalling Br J Cancer 2008 98 8 1348 1356 10.1038/sj.bjc.6604319 18362935 PMC2361714

[B12] Giles RH van Es JH Clevers H Caught up in a Wnt storm: Wnt signaling in cancer Biochim Biophys Acta 2003 1653 1 1 24 12781368 10.1016/s0304-419x(03)00005-2

[B13] Liu C Li Y Semenov M Han C Baeg GH Tan Y Zhang Z Lin X He X Control of beta-catenin phosphorylation/degradation by a dual-kinase mechanism Cell 2002 108 6 837 847 10.1016/S0092-8674(02)00685-2 11955436

[B14] Behrens J von Kries JP Kuhl M Bruhn L Wedlich D Grosschedl R Birchmeier W Functional interaction of beta-catenin with the transcription factor LEF-1 Nature 1996 382 6592 638 642 10.1038/382638a0 8757136

[B15] Tetsu O McCormick F Beta-catenin regulates expression of cyclin D1 in colon carcinoma cells Nature 1999 398 6726 422 426 10.1038/18884 10201372

[B16] Huang D Du X Crosstalk between tumor cells and microenvironment via Wnt pathway in colorectal cancer dissemination World J Gastroenterol 2008 14 12 1823 1827 10.3748/wjg.14.1823 18350618 PMC2700405

[B17] Brabletz T Jung A Reu S Porzner M Hlubek F Kunz-Schughart LA Knuechel R Kirchner T Variable beta-catenin expression in colorectal cancers indicates tumor progression driven by the tumor environment Proc Natl Acad Sci USA 2001 98 18 10356 10361 10.1073/pnas.171610498 11526241 PMC56965

[B18] Zhang B Ougolkov A Yamashita K Takahashi Y Mai M Minamoto T beta-Catenin and ras oncogenes detect most human colorectal cancer Clin Cancer Res 2003 9 8 3073 3079 12912959

[B19] Wong SC Lo ES Lee KC Chan JK Hsiao WL Prognostic and diagnostic significance of beta-catenin nuclear immunostaining in colorectal cancer Clin Cancer Res 2004 10 4 1401 1408 10.1158/1078-0432.CCR-0157-03 14977843

[B20] Yoon WH Jung YJ Kim TD Li G Park BJ Kim JY Lee YC Kim JM Park JI Park HD No ZS Lim K Hwang BD Kim YS Gabexate mesilate inhibits colon cancer growth, invasion, and metastasis by reducing matrix metalloproteinases and angiogenesis Clin Cancer Res 2004 10 13 4517 4526 10.1158/1078-0432.CCR-04-0084 15240544

[B21] Yoon WH Park HD Lim K Hwang BD Effect of O-glycosylated mucin on invasion and metastasis of HM7 human colon cancer cells Biochem Biophys Res Commun 1996 222 3 694 699 10.1006/bbrc.1996.0806 8651907

[B22] Kang DW MG Park do Y Hong KW Min do S Rebamipide-induced downregulation of phospholipase D inhibits inflammation and proliferation in gastric cancer cells Exp Mol Med 2010 42 8 555 564 10.3858/emm.2010.42.8.056 20625243 PMC2928928

[B23] Shin SYKYJ Song KS Kim NY Jeong SY Park JH Seo KS Heo JY Kwon HJ Park JI Park SK Kweon GR Yoon WH Hwang BD Lim K Mechanism of Anti-invasive action of Docosahexaenoic Acid in SW480 human colon cancer cell Journal of Life Science 2010 20 4 561 571 10.5352/JLS.2010.20.4.561

[B24] Bae DG Gho YS Yoon WH Chae CB Arginine-rich anti-vascular endothelial growth factor peptides inhibit tumor growth and metastasis by blocking angiogenesis J Biol Chem 2000 275 18 13588 13596 10.1074/jbc.275.18.13588 10788475

[B25] Alvarez OA Carmichael DF DeClerck YA Inhibition of collagenolytic activity and metastasis of tumor cells by a recombinant human tissue inhibitor of metalloproteinases J Natl Cancer Inst 1990 82 7 589 595 10.1093/jnci/82.7.589 2156082

[B26] Eunsook Nam CP Maspin Suppresses Survival of Lung Cancer Cells through Modulation of Akt Pathway Cancer Res Treat 2010 42 1 42 47 10.4143/crt.2010.42.1.42 20369051 PMC2848751

[B27] Clevers H Wnt/beta-catenin signaling in development and disease Cell 2006 127 3 469 480 10.1016/j.cell.2006.10.018 17081971

[B28] Henderson BR Nuclear-cytoplasmic shuttling of APC regulates beta-catenin subcellular localization and turnover Nat Cell Biol 2000 2 9 653 660 10.1038/35023605 10980707

[B29] Munemitsu S Albert I Souza B Rubinfeld B Polakis P Regulation of intracellular beta-catenin levels by the adenomatous polyposis coli (APC) tumor-suppressor protein Proc Natl Acad Sci USA 1995 92 7 3046 3050 10.1073/pnas.92.7.3046 7708772 PMC42356

[B30] Fearon ER Vogelstein B A genetic model for colorectal tumorigenesis Cell 1990 61 5 759 767 10.1016/0092-8674(90)90186-I 2188735

[B31] Calviello G Resci F Serini S Piccioni E Toesca A Boninsegna A Monego G Ranelletti FO Palozza P Docosahexaenoic acid induces proteasome-dependent degradation of beta-catenin, down-regulation of survivin and apoptosis in human colorectal cancer cells not expressing COX-2 Carcinogenesis 2007 28 6 1202 1209 10.1093/carcin/bgl254 17183061

[B32] Ougolkov AV Yamashita K Mai M Minamoto T Oncogenic beta-catenin and MMP-7 (matrilysin) cosegregate in late-stage clinical colon cancer Gastroenterology 2002 122 1 60 71 10.1053/gast.2002.30306 11781281

[B33] Baldus SE Monig SP Huxel S Landsberg S Hanisch FG Engelmann K Schneider PM Thiele J Holscher AH Dienes HP MUC1 and nuclear beta-catenin are coexpressed at the invasion front of colorectal carcinomas and are both correlated with tumor prognosis Clin Cancer Res 2004 10 8 2790 2796 10.1158/1078-0432.CCR-03-0163 15102686

[B34] Doyle JL Haas TL Differential role of beta-catenin in VEGF and histamine-induced MMP-2 production in microvascular endothelial cells J Cell Biochem 2009 107 2 272 283 10.1002/jcb.22123 19306293

[B35] Tamamura Y Otani T Kanatani N Koyama E Kitagaki J Komori T Yamada Y Costantini F Wakisaka S Pacifici M Iwamoto M Enomoto-Iwamoto M Developmental regulation of Wnt/beta-catenin signals is required for growth plate assembly, cartilage integrity, and endochondral ossification J Biol Chem 2005 280 19 19185 19195 10.1074/jbc.M414275200 15760903

[B36] Brabletz T Jung A Dag S Reu S Kirchner T beta-Catenin induces invasive growth by activating matrix metalloproteinases in colorectal carcinoma Verh Dtsch Ges Pathol 2000 84 175 181 11217438

[B37] Zhai Y Wu R Schwartz DR Darrah D Reed H Kolligs FT Nieman MT Fearon ER Cho KR Role of beta-catenin/T-cell factor-regulated genes in ovarian endometrioid adenocarcinomas Am J Pathol 2002 160 4 1229 1238 10.1016/S0002-9440(10)62550-3 11943708 PMC1867221

[B38] Egeblad M Werb Z New functions for the matrix metalloproteinases in cancer progression Nat Rev Cancer 2002 2 3 161 174 10.1038/nrc745 11990853

[B39] Sidenius N Blasi F The urokinase plasminogen activator system in cancer: recent advances and implication for prognosis and therapy Cancer Metastasis Rev 2003 22 2-3 205 222 12784997 10.1023/a:1023099415940

[B40] Kim GY Han MG Song YS Shin BC Shin YI Lee HJ Moon DO Lee CM Kwak JY Bae YS Lee JD Park YM Proteoglycan isolated from Phellinus linteus induces toll-like receptors 2- and 4-mediated maturation of murine dendritic cells via activation of ERK, p38, and NF-kappaB Biol Pharm Bull 2004 27 10 1656 1662 10.1248/bpb.27.1656 15467214

[B41] Park SK Kim GY Lim JY Kwak JY Bae YS Lee JD Oh YH Ahn SC Park YM Acidic polysaccharides isolated from Phellinus linteus induce phenotypic and functional maturation of murine dendritic cells Biochem Biophys Res Commun 2003 312 2 449 458 10.1016/j.bbrc.2003.10.136 14637158

[B42] Han SB Lee CW Kang JS Yoon YD Lee KH Lee K Park SK Kim HM Acidic polysaccharide from Phellinus linteus inhibits melanoma cell metastasis by blocking cell adhesion and invasion Int Immunopharmacol 2006 6 4 697 702 10.1016/j.intimp.2005.10.003 16504934

[B43] Fidler IJ Ellis LM The implications of angiogenesis for the biology and therapy of cancer metastasis Cell 1994 79 2 185 188 10.1016/0092-8674(94)90187-2 7525076

[B44] Song YS Kim SH Sa JH Jin C Lim CJ Park EH Anti-angiogenic, antioxidant and xanthine oxidase inhibition activities of the mushroom Phellinus linteus J Ethnopharmacol 2003 88 1 113 116 10.1016/S0378-8741(03)00178-8 12902060

